# A *Kallikrein 15 *(*KLK15*) single nucleotide polymorphism located close to a novel exon shows evidence of association with poor ovarian cancer survival

**DOI:** 10.1186/1471-2407-11-119

**Published:** 2011-04-01

**Authors:** Jyotsna Batra, Christina M Nagle, Tracy O'Mara, Melanie Higgins, Ying Dong, Olivia L Tan, Felicity Lose, Lene Marie Skeie, Srilakshmi Srinivasan, Kelly L Bolton, Honglin Song, Susan J Ramus, Simon A Gayther, Paul DP Pharoah, Mary-Anne Kedda, Amanda B Spurdle, Judith A Clements

**Affiliations:** 1 Australian Prostate Cancer Research centre-Queensland and Institute of Health and Biomedical Innovation, Queensland University of Technology, Kelvin Grove, Brisbane, Queensland, 4059, Australia; 2 Molecular Cancer Epidemiology Laboratory, Queensland Institute of Medical Research, 300 Herston Road, Herston, Brisbane, Queensland, 4006, Australia; 3 Gynaecological Cancer Group, Queensland Institute of Medical Research, 300 Herston Road, Herston, Brisbane, Queensland, 4006, Australia; 4 Cancer Research UK Department of Oncology, University of Cambridge, Strangeways Research Laboratory, Cambridge, UK; 5 Division of Cancer Epidemiology and Genetics, National Cancer Institute, Rockville, Maryland, USA; 6 Department of Gynecological Oncology, University College London, Elizabeth Garrett Anderson, Institute for Women's Health, University College London, London, UK

**Keywords:** Kallikrein 15, single nucleotide polymorphisms, ovarian cancer, splice variants

## Abstract

**Background:**

*KLK15 *over-expression is reported to be a significant predictor of reduced progression-free survival and overall survival in ovarian cancer. Our aim was to analyse the *KLK15 *gene for putative functional single nucleotide polymorphisms (SNPs) and assess the association of these and *KLK15 *HapMap tag SNPs with ovarian cancer survival.

**Results:**

*In silico *analysis was performed to identify *KLK15 *regulatory elements and to classify potentially functional SNPs in these regions. After SNP validation and identification by DNA sequencing of ovarian cancer cell lines and aggressive ovarian cancer patients, 9 SNPs were shortlisted and genotyped using the Sequenom iPLEX Mass Array platform in a cohort of Australian ovarian cancer patients (N = 319). In the Australian dataset we observed significantly worse survival for the *KLK15 *rs266851 SNP in a dominant model (Hazard Ratio (HR) 1.42, 95% CI 1.02-1.96). This association was observed in the same direction in two independent datasets, with a combined HR for the three studies of 1.16 (1.00-1.34). This SNP lies 15bp downstream of a novel exon and is predicted to be involved in mRNA splicing. The mutant allele is also predicted to abrogate an HSF-2 binding site.

**Conclusions:**

We provide evidence of association for the SNP rs266851 with ovarian cancer survival. Our results provide the impetus for downstream functional assays and additional independent validation studies to assess the role of *KLK15 *regulatory SNPs and *KLK15 *isoforms with alternative intracellular functional roles in ovarian cancer survival.

## Background

Ovarian cancer is an aggressive disease with high metastatic potential and is frequently diagnosed at an advanced stage [1,2]. *In vitro *studies show that malignant cells synthesize and secrete proteolytic enzymes which disrupt basement and extracellular membranes to allow malignant cells to invade neighboring tissues and metastasize [3]. Members of the Kallikrein-related (KLK) peptidase family are part of a proteolytic enzymatic cascade activated in aggressive forms of hormone-related cancers including ovarian cancer [4-6]. The KLKs are encoded by a 15-member gene family clustered together in a region of approximately 320 kb on chromosome 19q13.4 [5-7]. *KLK15 *(encoding for KLK15, previously reported as hK15, or prostinogen) is the most recently cloned member of the human kallikrein gene family [8], sharing a high degree of structural similarity with KLK3 (PSA), and is positioned adjacent to the *KLK3 *gene [5,8]. *KLK15 *mRNA expression levels are up-regulated in prostate cancer [9-11] and ovarian cancer [12]. In addition, *KLK15 *mRNA expression is reported to be a favorable diagnostic marker for breast cancer [13], and a significant predictor of reduced progression-free survival and overall survival after ovarian cancer diagnosis [12]. Several studies have been undertaken investigating the role of *KLK *genetic variants in different cancers, aiming to better understand cancer biology and also to identify potential new targets for genetic testing with regard to cancer risk and prognosis [14-17]. Of note, we have recently identified a PSA promoter SNP to be associated with ovarian cancer survival (O'Mara, manuscript submitted), however, no studies have been undertaken to assess the role of *KLK15 *genetic variation in ovarian cancer prognosis.

We used *in silico *approaches for data mining of the *KLK15 *gene for potential functional motifs and differential splicing to determine regions of the gene that could be functionally compromised by genetic variations. This was supplemented by identification of potentially functional common SNPs using database searches and re-sequencing of ovarian cancer cell lines and patients, to prioritize variants for ovarian cancer prognosis studies. We then assessed the association between ovarian cancer survival and 9 SNPs tagging 22 prioritized *KLK15 *SNPs in an Australian dataset, and undertook replication studies in two other ovarian cancer studies to validate our findings.

## Methods

### Study Participants and Genotyping

The initial phase of this study included 319 Australian women diagnosed with primary invasive epithelial ovarian cancer between 1985 and 1997. Over half of the women (N = 207, 65%) had participated in a large population-based case-control study of the etiology of ovarian cancer. Briefly, these women were ascertained through major gynecology-oncology treatment centres in the three most populous Australian states: Queensland, New SouthWales and Victoria. A central gynecologic histopathologist reviewed all pathology reports and sections of each tumor to confirm the diagnosis and histological subtype. The remaining women (N = 112, 35%) were ascertained as consecutive incident cases from the Royal Brisbane Hospital (RBH), Queensland, Australia. The patients in the study were included on the basis on availability of DNA and survival/follow up data. Information on diagnosis, disease stage (using the International Federation of Gynecologists and Obstetricians [FIGO] criteria), tumor histology and grade was abstracted retrospectively from the women's medical records and pathology reports or, for a subset of cases, from the RBH Gynecology Oncology database. Full details of the methods and the characteristics of the cases have been reported previously [15,18]. The women were followed for mortality using personal identifiers which were linked to the Australian National Death Index (NDI), State Cancer Registry records and the RBH Gynecology Oncology database. DNA was extracted as described in previous studies [15,18] and genotyping of the *KLK15 *SNPs was performed using the Sequenom iPLEX MassArray platform (San Diego, CA, USA) according to manufacturer instructions. The first replication sample set included 1815 patients from the stage 1 of an ovarian cancer genome-wide association study (GWAS), all with confirmed invasive epithelial ovarian cancer from four different ovarian cancer case series in the United Kingdom (UK): United Kingdom Ovarian Population study (UKOPS), Study of Epidemiology and Risk Factors in Cancer Heredity (SEARCH), Royal Marsden Hospital (RMH) and United Kingdom Familial Ovarian Cancer Registry (UKFOCR), as described previously [19]. Genotyping of the replication sample set was performed as part of the first published genome-wide association study (GWAS) of ovarian cancer [19].

The second replication dataset consisted of 413 women with invasive epithelial ovarian cancer from The Cancer Genome Atlas (TCGA) Pilot Project established by the National Cancer Institute and the National Human Genome Research Institute. Information about TCGA and the investigators and institutions that constitute the TCGA research network can be found at their website [20]. Genotyping and clinical data for ovarian cancer patients were downloaded via the TCGA data portal.

Progression-free survival, residual disease status and platinum sensitivity data were not available for the Australian or European sample sets. Informed consent was obtained from all the participating subjects. Human Ethics Committee approval was given for the recruitment and genotyping of all the individuals included in this study.

### Web based search for putative splice-variants

Access information for the *KLK15 *gene and its protein product was downloaded from different databases as given in Additional file 1. Potential *KLK15 *alternative splice variants were identified through sequence alignment analysis of the splice variants and Expression Sequence Tags (ESTs) reported in publicly available databases including NCBI and Ensembl. Sequence alignments were performed using the clustal W platform. SignalP 3.0 Server and PSORT II Prediction default matrices were used to determine if each *KLK15 *isoform contained a signal peptide. mRNA folding of all the *KLK15 *variants was analysed by *in silico *modeling using *mfold *version 2.3. Additional file 2 details the websites used for the *in silico *analysis.

### *In silico *Promoter and SNP analysis

The 11 kb region upstream of exon 1 on chromosome 19 (56020307 to 56037591 bp, NCBI build 36.3) along with the *KLK15 *coding sequence (Ensemble ID; ENSGG000000174562) was selected for putative promoter and SNP analysis using websites in Additional file 2. In brief, WWW Promoter Scan, PromoterInspector, CpG Islands, and Cister were used to predict and analyse putative promoter regions. Since hormonal steroids are known to be key regulators of *KLK *expression in hormone dependent cancers [21,22], we assessed the promoter regions for putative estrogen and androgen regulatory elements. Dragon ERE finder (sensitivity of 0.83) and Cister (ERE matrix) were used to find putative estrogen response elements (EREs). Cister (ARE matrix) and JASPAR (threshold 75%) were used to identify putative androgen response elements (AREs). SIFT and Polyphen were used to predict the functional significance of non-synonymous SNPs (nsSNPS). Webserver FastSNP was used for predicting the functional significance of the UTR and splice site SNPs. Analysis for putative microRNA (miRNA) sites was performed using miRBase Targets V4, Target scan, miRanda, PicTar and Patrocles.

### Cell culture, RT PCR and sequencing of *KLK15 *putative promoter region

The normal ovarian cell line HOSE17.1 and serous epithelial ovarian carcinoma cell lines OVCA432, SKOV3, PEO1 were cultured as previously described [15]. RNA extraction and reverse transcription-PCR (RT-PCR) were performed as previously published [15]. Briefly, RNA was isolated using the RNAeasy kit (Qiagen), treated with DNaseI (Invitrogen), and 2 μg RNA reverse-transcribed using Superscript III™ (Invitrogen). PCR was performed with primers (K15ExBFor, 5'-TTCAAGACCCCCAGATGGAGAAAAG-3' and K15Ex2Rev, 5'-CTTCCAGCAACTTGTCACCA-3') for 35 cycles with conditions of 94°C for 5 min followed by 35 cycles of 94°C, 64°C and 72°C for 1 min each, and a final extension at 72°C for 10 min. PCR for β2-microglobulin was used as an internal control. The PCR products were visualised by electrophoresis on 1.5% agarose gels stained with ethidium bromide. Genomic DNA was extracted using QIAamp DNA Mini Kit (Qiagen, Hilde, Germany). Four primer sets (Additional file 3) were designed using NETprimer http://www.premierbiosoft.com/netprimer/index.html to amplify segments of the putative *KLK15 *promoter region with the greatest density of likely functional SNPs, based on results from the *in silico *analysis. All reactions (20 uL) contained 10 ng of genomic DNA, 0.25 uM of each region specific primer (Proligo, Lismore, NSW, Australia), 0.2 mM dNTP mix (Roche, Castle Hill, NSW, Australia), 0.03 mM Magnesium Chloride (Invitrogen, Victoria, Australia). Denaturing agents DMSO and betaine (Sigma) were added to destabilize secondary structures in the template, and improve both the PCR and sequencing quality. PCR products were sequenced at the Australian Genome Research Facility (AGRF, Brisbane, Australia) using dideoxy dye chain-termination technology (Applied Biosystems). Sequences were aligned and analyzed using SeqMan™II (DNASTAR, WI, USA).

### Statistical Analysis

Survival time was calculated from date of diagnosis to date of death (from ovarian cancer) or censored at 1 September, 2004 or death from another cause. The Kaplan-Meier technique was used to estimate crude overall survival probabilities, and adjusted hazard ratios (HR) and 95% confidence intervals (CI) were obtained from Cox regression models adjusted for age group, FIGO stage, histologic subtype and grade. The TCGA dataset was adjusted for age, FIGO stage and histologic grade, but not subtype because all the samples were of the serous subtype. For the combined analysis, a weighted average of the log HR was calculated, taking into account random effects using the method of DerSimonian and Laird [23]. Linkage Disequilibrium (LD) maps were generated using Haploview 4.2 [24].

## Results

### Analysis of *KLK15 *transcripts

**Figure 1:**
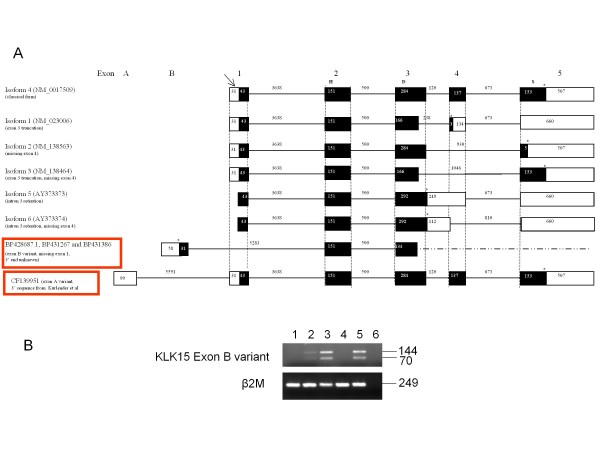
**Diagrammatic representation of human *KLK15 *mRNA transcripts and confirmation of the exon B variant by RT-PCR**. **(A) **Various isoforms (1-6) of *KLK15 *derived from the NCBI database are shown with the two new isoforms identified from the EST database containing novel exons, A and B, highlighted in red boxes. The unshaded boxes represent the noncoding exons, shaded black boxes for the isoforms represent the coding exons and the connecting lines the introns. Numbers inside boxes or above connecting lines represent exon or intron lengths in base pairs respectively. H, denotes histidine; S, denotes serine; and D, denotes aspartate [amino acids of the catalytic triad of the (putative) encoded proteins]. The arrowhead points to the common start codon and astericks (*) to the stop codon positions. **(B) **PCR was conducted on the cDNA from five different cell lines HOSE17.1 (1), PEO1 (2), OVCA432 (3), SKOV3 (4), LNCaP (5) and no cDNA (6) using the Forward primer in exon B and reverse primer in exon 2. Two products were obtained corresponding to with (144 bp) and without (70 bp) exon 1.

All of the published *KLK15 *mRNA variants [25] were collated from GenBank (Figure 1A). Six isoforms were observed. The classical form, isoform 4 (NM_017509), has five exons and encodes a protein of 256 amino acids (aa). However, this was recently extended to include an additional 5' untranslated exon (exon A) discovered from an EST clone (CF139951). Another 3 known isoforms (1-3) encode 3 different length proteins of 122aa, 161aa and 171aa respectively. Isoform 5 (AY373373) and isoform 6 (AY373374) both encode proteins of 162aa, since they share the same stop codon at the beginning of intron 3; the additional loss of exon 4 for isoform 6 does not affect protein length (Figure 1A). Isoform 5 was found to form the most stable secondary structure based on ΔG values predicted by mfold, while isoform 3 was the least stable (data not shown).

We also identified an additional *KLK15 *splice variant from the three new EST clones (GenBank accession No. BP428687, BP431267, BP431386). This new isoform has a novel exon between exon A and exon 1, that we have named exon B. Exon B is located 1575 bp upstream from exon 1, however all three EST clones show that exon B is spliced onto exon 2, skipping exon 1 (Figure 1A). The longest clone encodes a protein that is 106 aa long, shorter than that observed for the other isoforms; however it does not contain a stop codon and therefore is likely to be an incomplete sequence (Figure 1A). Using SignalP 3.0 Server and PSORT II, the exon B variant was not predicted to contain a possible cleavage site for an N-terminal signal peptide, thus is most likely to be an intracellular protein. In contrast, the other isoforms all are predicted to have signal peptides and a predominantly cytoplasmic and extracellular localization as expected for secreted proteins. The exon B variant was confirmed by RT-PCR in serous ovarian epithelial cancer cell lines PEO1 and OVCA432 and also in the prostate cancer LNCaP cell lines (Figure 1B).

### *In silico *promoter analysis to prioritize regions for sequencing and SNP modeling

**Figure 2:**
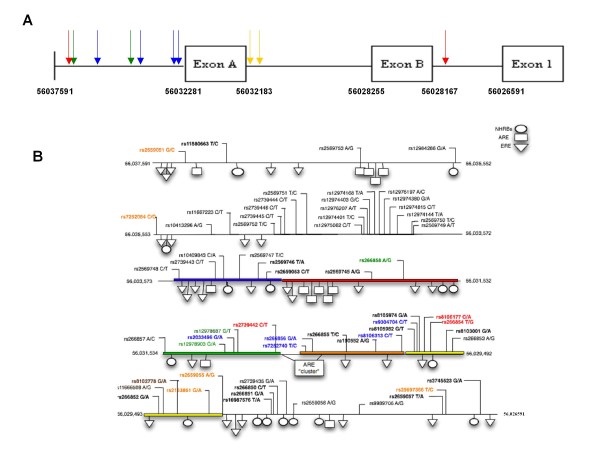
**The putative *KLK15 *promoter region**: **(A) **The 11 kb region upstream of exon 1 on chromosome 19 (56020307 to 56037591 bp, NCBI build 36.3) was downloaded for *in silico *analysis **(A) **schematic of the 5' region of the *KLK15 *gene with significant motifs noted: 2 putative Androgen Response Elements (AREs) were found by both JASPAR and Cister programs (red arrows); 4 putative Estrogen Response Elements (EREs) were found by both ERE finder and Cister programs (blue arrows); 2 putative cis-element clusters which indicate putative promoter regions found by Cister (green arrows); 2 putative promoter regions that overlap with CpG islands found by WWWPromoter Scan, Promoter Inspector and CpG Island Finder (yellow arrows) **(B) **AREs (boxes), EREs (triangle) and Nuclear Hormone Receptor Binding Sites (NHRBs) (circles) as predicted by individual softwares. The ARE "cluster" consists of 16 AREs. The colored bars (blue, red, green, orange, yellow) in the sequence indicate the 5 regions that were chosen for sequencing genomic DNA. The single nucleotide polymorphism (SNP)-modeling results are shown by color coding the functional SNPs validated in bold. Orange and red text coloring indicate a gain or loss of ERE motifs respectively, while green and blue indicate ARE gain or loss. The two brown SNPs indicate a loss of one ERE and a loss of two AREs. An additional four validated SNPs viz rs2659052, rs11880663, rs2659051, rs2659055 have been added to NCBI databases (not modeled here) from the time of our analysis.

Cister and JASPAR together identified 30 putative Androgen Response Elements (AREs) (15 upstream of exon A, 1 in exon A, 8 between exon A and B, 2 in exon B, and 4 between exon B and 1). Only 2 AREs (Figure 2A) were predicted by both tools (1 upstream of exon A and the other between exon B and exon 1). Dragon ERE finder and Cister predicted 30 putative Estrogen Response Elements (EREs) upstream of exon 1 (17 upstream exon A, 9 between exon A and B, and 4 between exon B and 1). Four EREs (Figure 2A) predicted by Dragon ERE finder overlapped an ERE predicted by Cister. Cister identified two clusters of cis-elements (Figure 2A), both upstream of exon A. The first cluster starts 4232 bp upstream of exon A, spans 467 bp and contains eight putative cis-elements. The second cluster starts 2108 bp upstream of exon A, spans 1892 bp and contains 10 putative cis-elements. Two putative promoter regions on the forward strand were detected using WWW Promoter Scan The first putative promoter is 189 bp downstream of exon A and spans 250 bp, and the second is 852 bp downstream of exon A and spans 252 bp. PromoterInspector detected one putative promoter region which is 992 bp downstream of exon A and spans 463 bp. CpGPlot showed three putative CpG islands with a percent C+G of above 50%, observed/expected ratio of above 0.60 and a length of over 100 bp: the first is 4519 bp upstream of exon A and spans 115 bp; the second is 538 bp downstream of exon A and spans 106 bp; the third is 778 bp downstream of exon A and spans 564 bp. The last two putative CpG islands overlap putative promoters found by WWW Promoter Scan and PromoterInspector (Figure 2A).

### SNP information and prediction of functional effects

#### Gene

A total of 76 (27 validated) SNPs in the exonic-intronic region of *KLK15 *were retrieved from the NCBI dbSNP database, comprising 10 exonic SNPs (4 non-synonymous, 1 synonymous and 5 in 3' UTR) (Additional file 4) and 66 intronic SNPs (data not shown). Although predicted to be damaging by FastSNP, SIFT and PolyPhen predicted that the validated non-synonymous (ns) exonic SNPs were unlikely to have any deleterious effect on protein structure (Additional file 4). FastSNP server predicted that the synonymous (s) SNP and the 3' UTR SNPs were not damaging with a risk score of 0-1 (Additional file 4). The rs3212852 SNP present at the 3' end of exon 3 was predicted to have a damaging effect with a medium-high risk score of 3-4. No damaging scores were observed for the additional 5 intronic SNPs located within 30 bp of exon-intron boundaries. We also predicted miRNA binding sites using three different software programs; Target Scan, miRanda and Patrocles. A maximum of 32 miRNA binding sites scattered throughout the gene were predicted by miRanda (data not shown). Interestingly, the 3'UTR SNP rs3212810 lies in the target sequence of mir-498, which has been reported to be dysregulated in prostate cancers [26]. Also, rs3212853 was predicted to be a potential polymorphic miRNA target site (using Patrocles target database), where the presence of the mutant allele creates a binding site for miR-193b (Additional file 4), shown to be involved in tumor progression and invasion in human breast cancer [27] and a tumor suppressor in prostate cancer [28].

#### Promoter

As gene expression may be influenced by promoter polymorphisms, we included analysis of SNPs in the 11 kb upstream region from exon 1 and found 62 (out of the total 101) validated SNPs. *In silico *analysis predicted that 8 SNPs created a new putative ERE/ARE site while 10 other SNPs were predicted to destroy one or more putative ERE/ARE sites using two different software programs; JASPAR and Cister for the ARE and Cistor and Dragon ERE finder for the ERE (Figure 2B). Further, it was observed that 22 SNPs created one or more new transcription factor binding sites (TFBS) while 25 SNPs completely lost one or more TFBS as predicted by Genomatrix ElDorado program (data not shown).

### Promoter sequencing of cancer cell lines and aggressive cancer patients

To confirm the polymorphic status of reported SNPs and identify novel SNPs with potential prognostic significance, we sequenced genomic DNA within the *KLK15 *promoter (Figure 2B) of four ovarian (normal -HOSE17.1; cancer- OVCA432, SKOV3 and PEO1) cell lines and the germline DNA of 30 ovarian cancer patients with aggressive disease. Sequencing analysis of the cell lines validated 15 SNPs, including 6 non-validated SNPs in the NCBI database (Additional file 5). Sequencing of genomic DNA from aggressive ovarian cancer patients validated additional 5 SNPs (Additional file 5), and identified two novel SNPs that were not predicted to be functionally relevant. Seven SNPs from the NCBI database were found to be non-polymorphic in our sequencing cohort. The putative functional role of the promoter SNPs is provided in Additional file 5.

### LD mapping and SNPs selection for survival studies

SNPs chosen for genotyping in this study were (i) identified as tagging SNPs using HapMap version 22 (April 07), using a minor allele frequency >0.05 and pairwise linkage disequilibrium threshold of r^2 ^> 0.8 (rs2659058, rs3212810, rs3745522, rs2659056, rs266851, rs2163861, rs266856), or (ii) chosen due to the above reported *in silico *prediction of functional effect on *KLK15 *expression (rs3212853, rs3212852, rs16987576, rs2659055, rs266853, rs266854, rs190552, rs266855, rs2739442, rs2033496, rs12978902, rs2659053, rs2569746, rs35711205, rs2569747) (Table 1). As the frequency data for many of these SNPs were not available, we genotyped all 22 SNPs in controls and generated the LD map using Haploview 4.2 (Batra et al, unpublished data; Additional File 6). All SNPs except rs3745522 were found to follow Hardy-Weinberg equilibrium (p < 0.01). SNP rs12978902 was non-polymorphic, while rs3212853, rs3212852, rs16987576 rs266853 and rs266854 were found to have minor allele frequencies < 0.05 (Table 1), so were not pursued further for the survival analysis. SNPs in high LD with other SNPs (r^2 ^> 0.9) were also not pursued further. Priority was given to putative functional SNPs, with a total of 12 SNPs shortlisted for further analysis (Table 1). Three of these SNPs (rs2659056, rs266855 and rs35711205) could not be genotyped in ovarian cancer samples because of assay incompatibilities.

**Table 1:** SNP selection for the survival studies.

SNP no	Name	Rationale	Position	ObsHET	PredHET	HWpval	MAF
1	**rs2659058**	**Tagged SNP **from HapMap	56017918	0.421	0.443	0.0869	0.331
2	**rs3212810**	**Tagged SNP **from HapMap and predicted to fall in miRNA binding site	56020548	0.364	0.369	0.6072	0.244
3	rs3212853	Ploymorphic miRNA target site	56020606	0.044	0.041	0.2863	0.037
4	rs3212852	Exon-Intron boundary	56021941	0.038	0.038	1	0.019
5	**rs3745522**	**Tagged SNP **from HapMap	56022744	0.349	0.383	0.0013	0.259
6	**rs2659056**	**Tagged SNP **from HapMap	56027755	0.383	0.382	1	0.257
7	**rs266851**	**Tagged SNP **from HapMap	56028151	0.297	0.306	0.2742	0.189
8	rs16987576	2 bp 3' of exon B, Imp for splicing	56028165	0.003	0.003	1	0.001
9	**rs2659055**	Predicted in silico to affect HRE	56029044	0.485	0.5	0.3033	0.49
10	rs2163861	**Tagged SNP **from HapMap	56029204	0.485	0.5	0.2841	0.489
11	rs266853	Predicted in silico to affect TFBS and HRE	56029520	0.001	0.001	1	0.001
12	rs266854	Predicted in silico to affect HRE	56029630	0.004	0.004	1	0.002
13	**rs190552**	Predicted in silico to affect TFBS and HRE	56030005	0.333	0.346	0.1823	0.222
14	**rs266855**	Predicted in silico to affect HRE	56030196	0.396	0.413	0.1259	0.292
15	rs266856	**Tagged SNP **from HapMap	56030318	0.333	0.349	0.1131	0.225
16	**rs2739442**	Predicted in silico to affect TFBS and HRE	56030985	0.479	0.493	0.2975	0.442
17	rs2033496	Predicted in silico to affect TFBS and HRE	56031019	0.49	0.493	0.8309	0.442
18	rs12978902	Predicted in silico to affect HRE	56031056	0	0	1	0
19	**rs2659053**	Predicted in silico to affect TFBS and HRE	56032606	0.449	0.472	0.0722	0.382
20	**rs2569746**	Predicted in silico to affect TFBS and HRE	56032727	0.462	0.487	0.0638	0.42
21	**rs35711205**	Novel SNP (reported later in NCBI)	56032818	0.296	0.309	0.1212	0.191
22	rs2569747	Predicted in silico to affect TFBS and HRE	56032875	0.459	0.487	0.0346	0.419

ObsHet: Observed heterozygous alleles; PredHET: Predicted heterozygous alleles; HWpval: Hardy-Weinberg equilibrium p value; MAF: Minor allele frequency; HRE: Hormone Response Element; TFBS: Transcription Factor Binding Site

SNPs in the *KLK15 *gene derived from the HapMap database and those by *in silico *prediction methods were genotyped in control individuals, and the minor allele frequency (MAF) and HWE were calculated using Haploview 4.2. SNPs in bold were shortlisted for genotyping and survival analysis on the basis on LD calculations.

### Association with ovarian cancer survival

Among the 319 Australian women with ovarian cancer, 188 (59%) died from the disease during the follow-up period, with a 5-year survival proportion of 45%. Selected clinical and pathologic characteristics of the Australian women and the women in the two independent datasets, the UK GWAS and TCGA, are shown in Table 2. Among the Australian women a little over three quarters were older than ≥50 years at diagnosis (77%), and many presented with late stage disease (71%), tumors of high grade (54%) and serous histological subtype (64%). In the UK GWAS dataset most women were ≥50 years at diagnosis (78%), just over half the group had early stage disease (56%) and serous histological subtype (54%). The TCGA dataset comprised women who were mostly older than ≥50 years at diagnosis (79%), and the majority had late stage (95%), high grade (88%) disease and all were of serous histologic subtype.

**Table 2:** Summary of clinical and pathological factors in the Australian, UK GWAS and TCGA ovarian cancer datasets.

	Australian dataset	UK GWAS dataset	TCGA dataset
	n* (%)	n* (%)	n* (%)
**Age group**			
<40	21 (7)	97 (5)	7 (2)
40-49	51 (16)	316 (17)	74 (19)
50-59	85 (26)	653 (36)	123 (31)
60-69	99 (31)	594 (33)	97 (24)
70+	63 (20)	155 (9)	96 (24)
**FIGO stage**			
I	54 (18)	586 (44)	8 (2)
II	35 (11)	162 (12)	12 (3)
III	192 (63)	507 (38)	312 (79)
IV	26 (8)	80 (6)	65 (16)
**Histological grade**			
well differentiated	41 (14)	239 (18)	2 (1)
moderately differentiated	94 (32)	418 (32)	44 (11)
poor/undifferentiated	156 (54)	643 (50)	344 (88)
**Histological subtype**			
serous	199 (64)	867 (54)	397 (100)
endometrioid	35 (11)	200 (13)	
mucinuous	23 (8)	320 (20)	
clear cell	21 (7)	173 (11)	
other	32 (10)	40 (2)	

*Numbers may not sum to total due to missing data.

SNP rs266851 showed statistically significant evidence for an association with worse survival (Table 3) in the Australian dataset. Increased risk was observed for the heterozygote genotype CT (HR 1.43, 95% CI 1.02-2.00), and also for the rare (2% of sample) homozygote genotype (HR 1.26, 95% CI 0.49-3.24); p trend = 0.01. Overall association under a dominant model was HR 1.42 with 95% CI = 1.02-1.96 (Table 3). This SNP was not found to be associated with ovarian cancer stage (p = 0.41) or grade (p = 0.39) (Table 2). None of the other SNPs investigated were associated with ovarian cancer survival in Australian dataset.

**Table 3:** Association between *KLK15 *Single Nucleotide Polymorphisms and ovarian cancer survival in Australian dataset

KLK15	n	n censored	Adjusted* HR (95% CI)	p value (trend)
***rs2659058***				
TT	123	74	1.0	
CT	138	88	1.13 (0.82-1.55)	0.75
CC	50	26	1.20 (0.75-1.91)	
***rs3212810***				
CC	207	122	1.0	
TC	87	51	1.01 (0.72-1.41)	0.29
TT	18	14	1.46 (0.82-2.62)	
***rs3745522***				
GG	187	106	1.0	
GT	81	48	0.97 (0.68-1.39)	0.15
TT	18	13	1.73 (0.93-3.24)	
***rs266851***				
CC	222	128	1.0	
CT	89	55	**1.43 (1.02-2.00)**	
TT	7	5	1.26 (0.49-3.24)	**0.01**
CT/TT	96	60	**1.42 (1.02-1.96)**	
***rs2659055***				
TT	84	52	1.0	0.23
TC	144	87	0.86 (0.60-1.23)	
CC	67	36	0.74 (0.47-1.17)	
***rs190552***				
TT	178	109	1.0	0.79
CT	114	65	1.04 (0.76-1.42)	
CC	19	13	1.21 (0.67-2.17)	
***rs2739442***				
GG	92	55	1.0	0.99
GA	133	76	1.09 (0.75-1.58)	
AA	89	54	1.08 (0.73-1.62)	
***rs2659053***				
GG	129	79	1.0	0.2
GA	118	73	0.93 (0.66-1.29)	
AA	63	34	0.95 (0.63-1.44)	
***rs2569746***				
AA	111	65	1.0	0.43
TA	119	74	0.90 (0.64-1.27)	
TT	57	36	1.12 (0.74-1.69)	

*Adjusted for age, FIGO stage, histological subtype, and grade.

In an attempt to replicate our results, we analyzed genotype data obtained from the UK GWAS and the TCGA datasets of ovarian cancer survival. We observed a similar direction of association in both the datasets for the dominant model (HR 1.07, 95% CI 0.94 -1.24 in UK GWAS data and HR 1.20, 95% CI 0.90-1.61 in TCGA data) (Table 4). Combining the results of these two studies (UK GWAS and TCGA data) with our data gave a summary HR of 1.16 (95% CI 1.0-1.35) for the SNP rs266851 under a dominant model (Table 4).

**Table 4:** Results of ovarian cancer survival analysis for the *KLK15 *rs266851 SNP in Australian, UK GWAS data, TCGA data and the combined datasets

*KLK15 *rs266851	n	n censored	Adjusted* HR (95% CI)*
***Australian***			
CC	222	128	1.0
CT/TT	96	60	1.42 (1.02-1.96)
			
UK GWAS**			
CC	1139	390	1.0
CT/TT	676	221	1.07 (0.94-1.24)
			
TCGA***			
CC	280	135	1.0
CT/TT	133	76	1.20 (0.90-1.61)
			
***Combined Analysis***			
CC	1641	653	1.0
CT/TT	905	357	1.16 (1.00-1.34)

*Adjusted for age, FIGO stage, histological subtype, and grade.

**The UK GWAS dataset was adjusted for age, FIGO stage, histologic subtype, grade and study site.

***The TCGA dataset was adjusted for age, FIGO stage and histologic grade, but not subtype as all the samples were of the serous subtype.

### Putative functional elements associated with SNP rs266851

The rs266851 SNP is predicted to have a FastSNP score = 0 in our initial analysis. As the FastSNP algorithm is weighted by the SNP position with respect to gene structure, and our data shows that rs266851 SNP is located 15 bp 3' of a novel exon B, we used ESEfinder [29] to predict the putative role of rs266851 SNP in mRNA splicing. ESEfinder prediction suggests loss of various SRp binding sites in the presence of T allele (Additional file 7), thus this SNP could be involved in alternative mRNA splicing.

Our preliminary unpublished data (Lai et al., personal communication) suggest that exon B could be an alternative transcription start site (data not shown). Thus, using the TFbind webtool [30], we predicted that the C to T change may abrogate the binding site for Heat Shock Factor 2 (HSF2), which activates expression of Heat Shock Proteins (HSP), which have been shown to play a vital role in tumorigenesis [31,32].

## Discussion

With the completion of the human genome project, an enormous amount of data has been generated and been made available to the research community through various database repositories. At the same time many bioinformatic/*in silico *tools have been made available to extract data and help conceptualize future studies. To our knowledge, this is the first study to use a comprehensive *in silico *approach for genetic data mining and regulatory region identification to direct SNP prioritization studies of the *KLK15 *gene and to analyze the association of *KLK15 *polymorphisms with ovarian cancer survival.

In our efforts to delineate the functional significance of the exonic SNPs, we used the Polyphen and SIFT algorithms which showed minimal associations of SNPs with likely functional amino-acid replacements. We thus proposed that SNPs in regulatory regions can act in *cis *or *trans*, by leading to gain or deletion of a TFBS. In order to identify the *KLK15 *promoter-regulatory region, multiple alignments of all the *KLK15 *splice variants and ESTs available in databases identified a novel isoform with a new exon (termed exon B), upstream of exon 1. This isoform utilizes exon B instead of exon 1 and is predicted to have a different function by encoding a protein lacking a signal peptide. Alternative *KLK *transcripts may possess both physiological and prognostic significance and some are emerging candidate biomarkers [25,33,34]. For example, the PSA-RP2 [35] and *KLK15 *isoform 3 [11,36] are upregulated in prostate cancer compared with benign prostatic hyperplasia tissues suggesting that differential mRNA splicing may be an important regulatory event in carcinogenesis. Thus, the novel exon B variant identified by this study needs to be explored further to assess its biological function and prognostic significance, and such studies are underway in our laboratory.

In order to assess the role of common genetic variation in altered regulation of *KLK15*, and with the knowledge that steroid hormones have been implicated in the etiology and/or progression of epithelial ovarian cancer [3], we analyzed the *in silico *recognized promoter regions for putative AREs and EREs. Though the results varied using different databases, overlapping predictions suggested the gain/loss of AREs and EREs in this region. We thus prioritized and confirmed the polymorphic status of the SNPs in this region by sequencing genomic DNA from four ovarian cell lines and 30 ovarian cancer patients. Of these putative functional and HapMap tag SNPs selected for genotyping, rs266851 was found to be associated with poor overall survival in ovarian cancer in the Australian cohort, The results were not found to be statistically significant in the TCGA and UK GWAS cohorts but were similar in magnitude and direction to that of Australian dataset. This is what might be expected for "winner's curse" phenomenon, where the estimated effect of a marker allele from the initial study reporting the association is often exaggerated relative to the estimated effect in follow-up studies. The difference in magnitude of risk estimates may also be partially explained by different case ascertainment criteria. In particular, follow-up of the large majority of the UK GWAS dataset was initiated at least 6 months after diagnosis through the cancer registry, biasing against recruitment of ovarian cancer cases with short-term survival after diagnosis, and resulting in a dataset that included more women with early FIGO stage disease (56% vs. 21% FIGO stage I and II) and fewer women diagnosed with serous ovarian cancer (47% vs. 66%). Consequently the 5-year survival rate was much higher in the UK GWAS dataset (77% compared to 45% for the Australian dataset), and it is interesting to note that the UK dataset showed the risk estimate of lowest magnitude (HR = 1.07). In contrast, the TCGA dataset with similar characteristics to the Australian dataset showed a risk estimate of greater magnitude (HR = 1.20), although still not as great as the initial risk estimate observed for the Australian dataset (HR 1.42). The overall pooled HR of 1.16 observed for the dominant model in our combined analysis is consistent with an association between rs266851 SNP and ovarian cancer survival in Australian dataset, and it would be important to confirm these findings in a larger sample set of incident cases. Interestingly, the rs266851 SNP was found to be associated with an increased risk of breast cancer in the Cancer Genetics Markers of Susceptibility (CGEMS) project Breast Cancer GWAS [37] with a trend p value = 0.008; heterozygote risk = 1.22 (1.05-1.41); homozygote risk = 1.49 (1.11-2.00) (cases N = 1145, controls N = 1142).

So far no other SNP has been found to be in complete LD with rs266851 from HapMap and next generation sequencing data. Using *in silico *analysis, we predicted that rs266851 could potentially be involved in differential mRNA splicing as the mutant allele was found to abrogate the binding site for the SRp55 and SF2/ASF splicing factors. There are several reports of elevated SR protein family expression associated with ovarian cancer [38] and SF2/ASF, specifically, has been described as a proto-oncogene [39]. We also propose that rs266851 might be involved in regulation of *KLK15 *transcription by altering the HSF-2 binding site. Recently an HSF-2 binding site has been detected in the *KLK5 *and *KLK7 *promoter-regulatory regions and their expression has been found to correlate with HSF-2 expression in microarray analysis of breast malignancies [40]. Nevertheless, more experimental evidence is required to understand the effects of this variant on *KLK15 *expression and/or splicing.

## Conclusions

This study supports a role of the *KLK15 *gene in ovarian cancer by providing suggestive evidence for an association of the rs266851 SNP with ovarian cancer survival. The location of this SNP adjacent to a novel exon B and in putative HSF2 and SRp binding sites should provide impetus for downstream functional assays and additional independent validation studies to assess the role of *KLK15 *regulatory SNPs and *KLK15 *isoforms with alternative intracellular functional roles in ovarian cancer survival. Our study also has applicability to studies investigating the role of *KLK15 *genetic variation in other hormone-related cancers, namely prostate, endometrial and breast cancer.

## Authors' contributions

JB organised the data, prepared the manuscript and performed sequencing and bioinformatic analyses; CMN performed statistical analyses; TOM performed the genotyping of Australian samples; MH, LMS, FL and SS helped in bioinformatic analysis and sequencing; YD and OLT performed RT PCR and sequence alignment; KB, HS, SR, SG and PP contributed to the production and analysis of the UK genome-wide association study; MAK, ABS and JAC conceived the study, participated in the design and coordination of the study; ABS and JAC helped to draft the manuscript. All authors read and approved the final manuscript.

## Pre-publication history

The pre-publication history for this paper can be accessed here:

http://www.biomedcentral.com/1471-2407/11/119/prepub

## Supplementary Material

Additional file 1 **Access information for *KLK15***. Information for *KLK15 *gene and its protein product was obtained from different databases and is outlined in the additional file1. Click here for file

Additional file 2 ***In silico *promoter and SNP analysis**. Additional file 2 details the different websites and software used to scan the promoter region of *KLK15 *and to predict the functional significance of single nucleotide polymorphisms in and around the gene. Click here for file

Additional file 3 **Primer sequences**. Additional file 3 details the primer sequence used for sequencing the *KLK15 *promoter region Click here for file

Additional file 4 ***KLK15 *intragenic SNPs and the results of *in silico *analysis**. Additional file 4 details the SNPs in *KLK15 *exon and exon Intron boundary and the results of *in silico *analysis on these SNPS. Click here for file

Additional file 5 **Validated *KLK15 *promoter SNPs**. SNPs validated by *KLK15 *promoter sequencing of genomic DNA from ovarian cell lines and aggressive patients and their predictive functional role. Click here for file

Additional file 6 **Linkage Disequilibrium map generated by Haploview 4.2**. Frequency data was generated for the control individuals and the LD map was plotted. SNPs not in bold were found to have frequencies < 0.05. Click here for file

Additional file 7 **Prediction of functional role of the rs266851 SNP**. ESE Finder Matrices analysis for SRp40, SC35, SF2/ASF and SRp55 splicing proteins. Click here for file
