# Supplementary material for: A Kallikrein 15 (KLK15) single nucleotide polymorphism located close to a novel exon shows evidence of association with poor ovarian cancer survival
Source: BMC Cancer. 2011 Apr 1;11:119. doi: 10.1186/1471-2407-11-119 (PMC3080344; doi:10.1186/1471-2407-11-119)
Supplement: Additional file 1 — Access information for KLK15. Information for KLK15 gene and its protein product was obtained from different databases and is outlined in the additional file1. [file 1471-2407-11-119-S1.DOC]

**Supplementary Table 1. Access information for the *KLK15* gene and its protein product** obtained from different databases.

| ***KLK15* Gene** |  |  |
| --- | --- | --- |
|  |  |  |
| Gene name | human tissue kallikrein gene 15 |  |
| Official symbol | KLK15 (gene), hK15 (protein) |  |
| Synonyms | ACO; HSRNASPH; HGNC:20453; ACO protease; kallikrein-like serine protease; prostinogen; prostin |  |
| GenBank accession No. | AF242195 (genomic) | GenBank |
|  | NM 107509 (mRNA) | (http://www.ncbi.nlm.nih.gov/entrez/ |
|  | NP_059979 (protein) | query.fcgi?db=Nucleotide) |
| UniGene Cluster | Hs.250770 | Unigene (http://www.ncbi.nlm.nih.gov/entrez/ query.fcgi?db=unigene) |
| UCSC | X75363 (mRNA); Q9H2R5 (protein) | UCSC (http://genome.ucsc.edu) |
| GeneCards ID | GC19M056020 | GeneCard (http://bioinfo.weizmann.ac.il/cards/index.shtml) |
| Genew ID | HGNC:20453; KLK15 | Genew (http://www.gene.ucl.ac.uk/nomenclature/) |
| CleanEx ID | HGNC:20453; KLK15 | CleanEx (http://www.cleanex.isb-sib.ch/) |
| GeneLynx ID | 6578 | GeneLynx (http://www.genelynx.org/) |
| GenATlas ID | kallikrein 15 | GeneAtlas (http://www.geneatlas.org/ ) |
| GO ID | GO:0004252 | GO (http://www.geneontology.org/) |
| SOURCE ID | KLK15; *Homo sapiens* | SOURCE(http://genome-www.stanford.edu/listeria/gut/ ) |
| BLOCKS ID | Q9H2R5. |  |
| Ensembl Gene ID | ENSG00000174562 | Ensembl (http://www.ensembl.org/) |
| Gene ID | 55554 | Entrez Gene (http://www.ncbi.nlm.nih.gov/gquery/ gquery.fcgi) |
| Locus ID | HGNC:20453 | Entrez Gene (http://www.ncbi.nlm.nih.gov/gquery/ gquery.fcgi) |
|  |  |  |
| *hK15 protein* |  |  |
| Swiss-Prot/UniProt | Q9H2R5 | Swiss-Prot (http://au.expasy.org/sprot/) |
| MEROPS ID | S01.081 | MEROPS (http://merops.sanger.ac.uk/) |
| Isoelectric point | 8.0278 | Ensembl |
| Molecular weight, kDa | 28.087 | Swiss-Prot |
| Signal Peptide, aa | 15 | Swiss-Prot |
| Activating peptide, aa | 5 | Swiss-Prot |
| Mature protein, aa | 256 | Swiss-Prot |
| Cystein residues in mature enyme, aa | 12 |  |
| Substrate specificity | chymotrypsin-like |  |
