# Supplementary material for: A Kallikrein 15 (KLK15) single nucleotide polymorphism located close to a novel exon shows evidence of association with poor ovarian cancer survival
Source: BMC Cancer. 2011 Apr 1;11:119. doi: 10.1186/1471-2407-11-119 (PMC3080344; doi:10.1186/1471-2407-11-119)
Supplement: Additional file 2 — In silico promoter and SNP analysis. Additional file 2 details the different websites and software used to scan the promoter region of KLK15 and to predict the functional significance of single nucleotide polymorphisms in and around the gene. [file 1471-2407-11-119-S2.DOC]

**Supplementary Table 2: Websites for promoter and SNP analysis.** The *KLK15* gene sequence along with 11kb upstream of the first coding exon was obtained from Ensembl (Ensembl ID; ENSGG000000174562) for the putative promoter and SNP analysis using the following websites

| **Database** | **Website address** |
| --- | --- |
| clustal W platform | <http://www.ebi.ac.uk/Tools/clustalw/index.html>? |
| SignalP 3.0 Server | http://www.cbs.dtu.dk/services/SignalP/ |
| PSORT II Prediction | <http://psort.ims.u-tokyo.ac.jp/form2.html> |
| mfold | *mfold http://mfold.bioinfo.rpi.edu/* |
| Dragon ERE finder version 2 | http://sdmc.lit.org.sg/ERE-V2/index |
| Cister | http://zlab.bu.edu/~mfrith/cister.shtml |
| Promoter Scan Version 1.7 | http://www-bimas.cit.nih.gov/molbio/proscan/ |
| CpG Islands | http://www.ebi.ac.uk/Tools/emboss/cpgplot/index.html |
| ElDorado and PromoterInspector | http://www.genomatix.de/en/index.html |
| TRANSFAC | http://www.biobase-international.com/index.php?id=transfac |
| NHR Scan | http://www.cisreg.ca/cgi-bin/NHR-scan/nhr_scan.cgi |
| JASPAR | <http://jaspar.cgb.ki.se/cgi-bin/jaspar_db.pl> |
| SIFT, sequence homology based method | <http://blocks.fhcrc.org/sift/SIFT.html> |
| PolyPhen, structure-homology based method | <http://coot.embl.de/PolyPhen/> |
| FastSNP server | [http://fastsnp.ibms.sinica.edu.tw](http://fastsnp.ibms.sinica.edu.tw/) |
| ESEfinder | http://rulai.cshl.edu/tools/ESE/ |
| Target scan | http://www.targetscan.org/ |
| miRanda | http://cbio.mskcc.org/research/sander/data/miRNA2003/miranda_new.html |
| PicTar | <http://www.pictar.org/> |
| Patrocles | <http://www.patrocles.org/> |
