# Supplementary material for: A Kallikrein 15 (KLK15) single nucleotide polymorphism located close to a novel exon shows evidence of association with poor ovarian cancer survival
Source: BMC Cancer. 2011 Apr 1;11:119. doi: 10.1186/1471-2407-11-119 (PMC3080344; doi:10.1186/1471-2407-11-119)
Supplement: Additional file 3 — Primer sequences. Additional file 3 details the primer sequence used for sequencing the KLK15 promoter region [file 1471-2407-11-119-S3.DOC]

**Supplementary Table 3: PCR and sequencing primer information.** The primer sets were designed using NETprimer. The size of the sense and antisense primers, PCR product and their annealing temperature is given below

| ***KLK15*** | **PCR and Sequencing Primer sequences *** | **Size** | **Annealing temperature** | **Product size** |
| --- | --- | --- | --- | --- |
| **SNPset1** | [s] 5` TCT GGG AGA TCA AAA CAG TGG 3`  [as] 5` GCA GGT GTG CGA ATC ATA AA 3` | 21 bp  20 bp | 60 °C  60 °C | 749 bp |
| **SNPset2** | [s] 5` CTG CCC CTG AGA TGA AGA AC 3`  [as] 5` GTG GTG ACG AGG GAA AGG AG 3` | 20 bp  20 bp | 60 °C  62 °C | 699 bp |
| **SNPset3** | [s] 5` GTC CTC CAC TGC CAG AAT GT 3`  [as] 5` TGC ATT GAA AAC CTG GAC TG 3` | 20 bp  20 bp | 59 °C  60 °C | 666 bp |
| **SNPset4** | [s] 5` GGT TTT CAA TGC AAA AAC AGA 3`  [as] 5` CTT GGG GCA GAG TCC TGG AT 3` | 21 bp  20 bp | 57 °C  61 °C | 770 bp |

* [s] – sense, [as] – antisense, bp= base pairs
