# Supplementary figures and images for: A Kallikrein 15 (KLK15) single nucleotide polymorphism located close to a novel exon shows evidence of association with poor ovarian cancer survival
Source: BMC Cancer. 2011 Apr 1;11:119. doi: 10.1186/1471-2407-11-119 (PMC3080344; doi:10.1186/1471-2407-11-119)

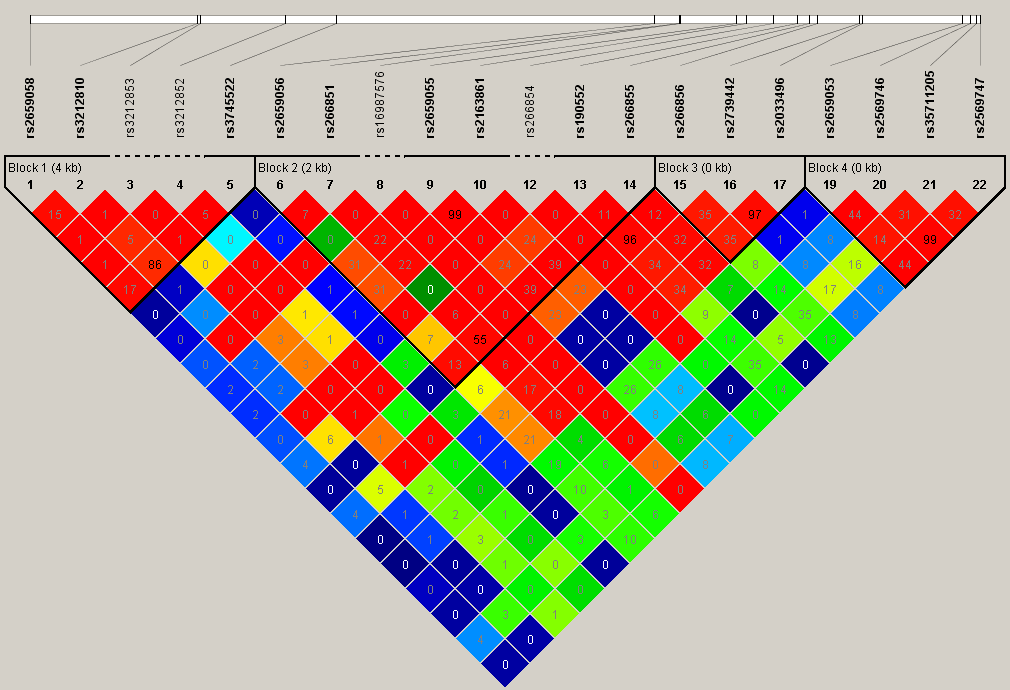

Supplement: Additional file 6 — Linkage Disequilibrium map generated by Haploview 4.2. Frequency data was generated for the control individuals and the LD map was plotted. SNPs not in bold were found to have frequencies < 0.05. [file 1471-2407-11-119-S6.PNG]
